# A Model Parlor Magazine

**Published:** 1873-01

**Authors:** 


*A Model Parlor Magazine.*

This is essentially true of Demorest's Monthly, which combines literary attractions of a very high order, with the most complete array of reliable fashions of any periodical in the country. It is a "model," also, of artistic beauty in its illustrations and typography, as any one can see by reference to the beautiful February number, which we find on our table. This popular magazine, together with two beautiful and artistic oil chromos, representing in value thirteen dollars, and all for three dollars, is among the marvels in literary enterprises.

Book Notices are crowded out of this number.

